# Listening to Worries: A Phenomenological Inquiry Into Open Dialogue in Community Settings

**DOI:** 10.7759/cureus.96249

**Published:** 2025-11-06

**Authors:** Daisuke Son, Mihoko Tsukahara

**Affiliations:** 1 Department of Community-Based Family Medicine, Faculty of Medicine, Tottori University, Yonago, JPN; 2 Department of Medical Education Studies, International Research Center for Medical Education, Graduate School of Medicine, The University of Tokyo, Tokyo, JPN; 3 Department of Psychiatry, Beruf, Tokyo, JPN

**Keywords:** community dialogue, interpretative phenomenological analysis, open dialogue, professional identity, psychological safety, self-reflection, social connectedness

## Abstract

Background: Open Dialogue, a therapeutic approach from Finland, emphasizes dialogical interactions to support individuals with psychiatric conditions. While its clinical effectiveness is well-documented, its use in broader community contexts remains limited. In Japan, the Machiken Dialogue adapts Open Dialogue to address everyday concerns in local settings. This study explores participants’ experiences with Machiken Dialogue and its potential to enhance social connectedness and emotional well-being.

Methods: A qualitative phenomenological analysis was conducted on 134 free-text responses from general citizens (n = 77) and healthcare, welfare, and care professionals (n = 57) who participated in sessions from 2017 to 2019. Interpretative phenomenological analysis was used to identify key experiential themes.

Results: Citizens emphasized psychological safety, self-discovery, and the importance of silence and multiple perspectives. Self-disclosure, though challenging, fostered connection and shared understanding. Professionals reported a shift from expert roles to co-participants, experiencing both liberation and difficulty maintaining neutrality. Balancing structured facilitation with open dialogue reflected ongoing tensions between clinical norms and dialogical values.

Conclusion: Machiken Dialogue offers a supportive space for self-expression, reflection, and empathy, suggesting its value in strengthening community ties and emotional connectedness. Its application beyond psychiatric care shows promise in fostering reflective communication and empathy. Nonetheless, challenges around facilitation, self-disclosure, and shifting professional roles point to the need for further research on effective community-based dialogical practices.

## Introduction

Open Dialogue is a therapeutic approach developed in Finland in the 1980s to support individuals with psychiatric disorders, particularly those experiencing acute psychotic episodes such as first-episode psychosis, schizoaffective disorder, and schizophrenia-spectrum conditions. Initially implemented in Western Lapland, it has been shown to reduce hospitalization rates and improve functional outcomes among patients with early psychosis [1-3]. Developed by Seikkula and colleagues, Open Dialogue is a network-oriented, dialogical process involving multiple healthcare professionals - typically including psychiatrists, nurses, psychologists, and social workers - who meet repeatedly with patients and their families to foster collaborative problem-solving through conversation [1,4,5]. Emphasizing early intervention, shared decision-making, and open-ended dialogue, this approach has demonstrated improved long-term recovery outcomes, fewer hospitalizations, and reduced reliance on psychotropic medication [1,2].

Over time, Open Dialogue has evolved beyond clinical psychiatric care, inspiring adaptations that address a broader range of social and personal challenges. One such adaptation is Anticipation Dialogue, a welfare-oriented approach that encourages future-focused discussions to alleviate everyday worries [6]. This extension reflects Open Dialogue’s emphasis on fostering trust, creating shared meaning, and enhancing social connectedness, all of which contribute to emotional resilience and collective well-being [7]. The dialogical framework has been explored in various contexts, including education, social work, and community development, demonstrating its potential to facilitate inclusive and reflective conversations that help individuals navigate uncertainty and change [8].

In Japan, Open Dialogue has gained increasing attention in psychiatric care, yet its application to general community worries remains limited [9]. To address this gap, the Machiken Dialogue was launched in 2017 as an initiative that applies the principles of Open Dialogue to everyday worries in the community [10]. This initiative creates structured yet open conversational spaces where citizens can engage in meaningful discussions about their concerns, fostering mutual understanding and social support. The Machiken Dialogue is particularly grounded in three core principles of Open Dialogue: the social network perspective, which emphasizes the inclusion of broader social connections; tolerance of uncertainty, which allows discussions to unfold organically without premature conclusions; and dialogism, which ensures that all voices in the conversation are acknowledged and actively engaged. These principles are central components of the Open Dialogue framework as described by Seikkula and Arnkil (2018) and further operationalized in Olson et al.’s (2014) fidelity criteria for dialogic practice [4,11]. They also resonate with Mikhail Bakhtin’s concept of polyphony, where multiple perspectives coexist without a single dominant narrative [12].

The social significance of Open Dialogue extends beyond individual psychological well-being to fostering social connectedness. Studies suggest that dialogical approaches strengthen social capital by creating networks of shared understanding and support, thereby enhancing individuals' ability to cope with adversity [11]. By encouraging active listening, self-reflection, and mutual validation, Open Dialogue has the potential to mitigate social isolation and promote a sense of community belonging [13]. Furthermore, this approach aligns with contemporary movements advocating for patient-centered care and deliberative democracy, emphasizing that decision-making - whether in healthcare or social policy - should be a participatory process grounded in dialogue and reciprocity [14,15].

Despite its theoretical potential, there is still limited empirical research on how Open Dialogue functions in non-clinical, community-based settings. This study aims to explore how participants experience the Machiken Dialogue and to examine the essential structure of their engagement in this setting. Specifically, it investigates how general citizens and professionals in healthcare, welfare, and care services perceive and reflect on their participation.

To achieve this, we conducted a qualitative phenomenological study based on post-session online surveys completed by both citizens and professionals who participated in the Machiken Dialogue sessions. Using an Interpretative Phenomenological Analysis (IPA) framework, we examined participants’ written reflections to uncover the lived experiences and key experiential themes that characterized their engagement in dialogical encounters. This approach allowed us to interpret how participants made sense of their experiences and what meanings they attributed to them. Through this analysis, we aimed to understand how structured dialogue initiatives contribute to personal insight, social connectedness, and professional reflection, thereby offering evidence for the broader applicability of Open Dialogue principles in promoting community resilience and emotional well-being beyond traditional psychiatric contexts.

## Materials and methods

The Machiken Dialogue: applying open dialogue to community worries

Since 2017, we have developed and implemented the Machiken Dialogue, an initiative that applies the principles of Open Dialogue to address the everyday worries of community members. Open Dialogue, originally developed in Finland in the 1980s, is a therapeutic approach designed to support individuals with psychiatric disorders, particularly those experiencing delusions [11]. This approach typically involves a multidisciplinary team - usually including psychiatrists, nurses, psychologists, and social workers - who engage in repeated dialogue meetings with a single patient and their family members. Each meeting generally lasts 60 to 90 minutes and is held several times a week during the acute phase, with the frequency gradually decreasing as the patient’s condition stabilizes. Over the course of treatment, patients and families may participate in multiple sessions depending on clinical needs and recovery progress [4,16]. Over time, Open Dialogue has been adapted beyond clinical settings, leading to the development of Anticipation Dialogue, a welfare-oriented application that encourages future-focused discussions to alleviate everyday worries [11]. 

While Open Dialogue has recently gained attention in Japanese psychiatric settings, its application to general community worries remains limited. The Machiken Dialogue extends this approach beyond the clinical domain by creating structured dialogues centered on citizens' everyday worries [10]. Participants are recruited from the local community, and the dialogue sessions are facilitated by trained moderators. Each session lasts 60 to 90 minutes and includes reflecting conversations, during which facilitators discuss their perspectives among themselves [17]. Topics addressed in past sessions include bereavement, chronic illness, employment challenges, and issues related to children and education. In some cases, follow-up dialogues are conducted with the same participants. This initiative aims to explore the potential of Open Dialogue as a tool for enhancing social support and fostering community resilience.

Participants and data collection

This study employed a qualitative phenomenological design using interpretative analysis to explore participants’ lived experiences of the Machiken Dialogue. Between May 2017 and September 2019, a total of 22 Machiken Dialogue sessions were conducted. Each session lasted 60 to 90 minutes and was held as a group meeting that included community members and trained facilitators. Two to three facilitators - typically physicians, nurses, and clinical psychologists - led each session. Participants were different for each session, and each individual attended only once. After each session, participants were invited to complete an online survey designed to gather qualitative data on their experiences.

The survey consisted of two open-ended questions: (1) What did you feel as a result of participating in the dialogue? and (2) In retrospect, what do you think you learned from the experience? 

These questions were developed by the research team and reviewed by two experts in qualitative research and dialogical practice to ensure their relevance, credibility, and clarity. Participants were encouraged to provide free-text responses based on their personal reflections. Demographic information such as age, gender, and occupation was not collected as part of this survey in order to ensure participants’ anonymity and encourage open reflection on their experiences.

The study employed a convenience sampling technique, as participation in the survey was voluntary among individuals who attended the Machiken Dialogue sessions. Data were collected through a written online survey consisting of open-ended questions rather than interviews, allowing participants to respond in their own time and words.

Inclusion and exclusion criteria

All individuals who participated in the Machiken Dialogue sessions between May 2017 and September 2019 were eligible to take part in the survey. Participation was entirely voluntary, and only those who provided informed consent by submitting their responses were included in the analysis. Incomplete or blank responses were excluded.

Data analysis

This study employed a phenomenological approach, using interpretative analysis to explore participants' lived experiences as expressed in their open-ended questionnaire responses [18,19]. The analysis followed an interpretative phenomenological analysis to uncover the underlying meanings embedded in participants’ narratives.

IPA is grounded in three theoretical pillars: phenomenology, hermeneutics, and idiography, which collectively shape its approach to understanding human experience in qualitative psychology [18,19]. Phenomenology, derived from Edmund Husserl’s eidetic method, focuses on how phenomena appear in individuals’ experiences, aiming to identify essential components through eidetic reduction. This involves bracketing preconceptions to let phenomena reveal themselves, prioritizing participants’ perceptions over predefined categories [18,19]. Martin Heidegger’s hermeneutic extension builds on this, emphasizing interpretation as a means to comprehend existence itself [20]. In IPA, researchers act as interpreters, akin to Hermes in mythology, translating participants’ experiences into meaningful insights while acknowledging their active role in the process [19]. This dual hermeneutic process involves participants interpreting their world and researchers decoding those interpretations, often asking critical questions like, *"What is the participant trying to achieve?"* or *"Is there an unintended meaning here?"* [19]. Idiography, the third pillar, underscores IPA’s commitment to in-depth, case-by-case analysis, contrasting with nomothetic approaches that seek universal laws [21]. IPA examines individual perspectives within unique contexts before deriving broader themes, ensuring a focus on the particular rather than the general. This synthesis renders IPA both descriptive - capturing how experiences manifest - and interpretative, recognizing that no phenomenon exists without interpretation. Practically, this framework supports IPA’s methodology by guiding researchers to explore subjective meaning-making through detailed, personal accounts, fostering a dynamic interplay between participant and researcher perspectives [18,19,22].

The process of IPA began with organizing and understanding the descriptions, in which all responses were carefully read multiple times to ensure familiarity with the data and to grasp the nuances of participants' experiences. Initial notes were made to highlight significant expressions, recurring concepts, and underlying meanings. Next, key themes were identified by recognizing patterns across individual responses. Through iterative reading, emerging themes were extracted and categorized into broader conceptual groups while ensuring that each response retained its individuality. Finally, structuring the essential experience involved examining relationships between themes to develop a coherent framework that captured the core meanings of participants’ experiences. A double hermeneutic process was applied, whereby participants' interpretations of their own experiences were analyzed to uncover deeper meanings and contextual influences.

Through this phenomenological approach, the study aimed to provide a nuanced understanding of participants' lived experiences as conveyed through their written reflections.

Ethical considerations

To protect participants' privacy and personal information, all questionnaire responses were anonymized. Consent for participation was considered given upon submission of the questionnaire; however, participation was entirely voluntary, and respondents had the freedom to choose whether to answer each question. The study was conducted in accordance with the Declaration of Helsinki (World Medical Association, 2013) and adhered to ethical guidelines for research involving human participants. This study was approved by the Ethics Review Committee of the University of Tokyo School of Medicine (Approval No. 10994).

## Results

This study analyzed a total of 134 responses, with contributions from both general citizens and professionals in healthcare, welfare, and care services. Of these, 77 responses were from citizens, and 57 were from professionals, mainly including physicians, nurses, clinical psychologists, occupational therapists, and welfare or care workers. Detailed demographic information such as age, gender, and occupation was not collected to protect anonymity and encourage participants to respond freely and reflectively. Therefore, no further demographic table is presented. An IPA was conducted to explore the participants' lived experiences and the essential structure of their engagement in the Machiken Dialogue sessions.

The key themes and representative quotations from both citizens and professionals are presented narratively in the text to preserve the richness and context of participants’ expressions. While a table format was considered, a narrative presentation was chosen to convey the nuances of meaning and dialogical interactions more faithfully, consistent with the interpretative phenomenological approach adopted in this study.

Phenomenological qualitative analysis of citizens' free responses

Identifying Key Themes (Citizens)

The responses of general citizens revealed several recurring themes that shaped their experiences, including a sense of safety and comfort, self-discovery through dialogue, the multiplicity of voices and empathy, the importance of language and silence, the impact of reflecting (reflexivity), the challenges and significance of self-disclosure, and the role and challenges of facilitation.

Sense of safety and comfort: Many participants emphasized the importance of a secure and accepting space, which allowed them to express themselves freely.

"I felt at ease. I had a sense of reassurance that I could talk without fear."

"The atmosphere was warm and accepting, which made me comfortable sharing my thoughts."

Self-discovery through dialogue: Engagement in dialogue prompted participants to reflect on their emotions, biases, and perspectives, leading to new insights about themselves.

"I realized my own immaturity, but that in itself was a meaningful realization."

"By honestly sharing my thoughts, I was able to observe my emotions more objectively."

Multiplicity of voices and empathy: Participants noted that exposure to diverse perspectives expanded their understanding and deepened their empathy.

"Hearing different interpretations of the same story made me appreciate how unique each perspective is."

"I started to notice how others’ perceptions differed from mine, which broadened my understanding."

Importance of language and silence: Many participants reflected on the difficulty of choosing words carefully in a non-judgmental space, as well as the role of silence in communication.

"I was hesitant to speak because I wanted to avoid negating others’ viewpoints."

"Initially, silence felt uncomfortable, but I later realized its significance in the conversation."

Impact of reflecting (reflexivity): The practice of reflecting - where participants or facilitators restated and processed what was said - was found to be transformative.

"Through reflection, I began to view the discussion from a more detached perspective."

"Repeated reflection allowed me to truly ‘hear’ what others were saying."

Challenges and significance of self-disclosure: While some found self-disclosure difficult, those who shared their experiences often reported a sense of relief and validation.

"At first, I regretted sharing too much, but later, I felt relieved when others resonated with my experiences."

"Hearing others share their experiences encouraged me to open up as well."

Role and challenges of facilitation: Participants acknowledged the crucial role of facilitators and the difficulty of guiding discussions without imposing structure or direction.

"The facilitator’s ability to speak naturally helped create a sense of unity in the group."

"I realized how challenging it is to ask open-ended questions without leading the speaker."

Structuring the Essential Experience (Citizens)

For general citizens, the core experience of Open Dialogue was grounded in the establishment of a safe and non-judgmental environment that allowed for authentic self-expression. Within this space, participants engaged in verbal reflection and attentive listening, which facilitated a deeper understanding of themselves and others. As they shared their thoughts and heard diverse perspectives, many discovered previously unrecognized aspects of their inner lives. This multiplicity of voices fostered empathy and broadened their worldview, helping them to connect more meaningfully with others. Silence, often initially perceived as awkward, came to be valued as a space for reflection and emotional processing, shaping the rhythm and depth of the dialogue. Participants also highlighted the complexity of self-disclosure - though often accompanied by initial hesitation, revealing personal experiences proved to be both challenging and rewarding, leading to feelings of connection and validation. Central to this experience was the role of the facilitator, who navigated the delicate balance between neutrality and gentle guidance, enabling a space where dialogue could unfold organically within an atmosphere of mutual respect and psychological safety.

Phenomenological qualitative analysis of professionals' free responses

Identifying Key Themes (Professionals)

Similarly, key themes that emerged from professionals' responses included the transformative nature of Open Dialogue, the role and professional identity in dialogue, challenges in maintaining neutrality and non-directiveness, the impact of polyphony (multiplicity of voices), the personal emotional impact and self-reflection, tensions between clinical thinking and Open Dialogue, and facilitation challenges and structural considerations.

The transformative nature of open dialogue: Many professionals noted that Open Dialogue felt distinct from conventional medical or therapeutic discussions.

"Unlike typical medical meetings, the conversation naturally flowed, creating a unique rhythm." (Physician)

"The experience demonstrated how Open Dialogue shifts individuals from isolated thinking into a collective meaning-making process." (Occupational Therapist)

Role and professional identity in dialogue: Some professionals felt liberated by stepping away from their expert roles, while others were concerned about how their identity was perceived.

"I enjoyed engaging as a person rather than as a professional." (Occupational Therapist)

"I worried about unintentionally guiding the conversation from a medical standpoint." (Nurse)

Challenges in maintaining neutrality and non-directiveness: Resisting the urge to provide solutions was a challenge for many professionals.

"I struggled to suppress my instinct to analyze and solve problems." (Clinical Psychologist)

"I found it difficult to avoid directing the discussion towards a resolution." (Occupational Therapist)

Impact of polyphony (multiplicity of voices): Listening to multiple perspectives was both enriching and challenging.

"A new voice emerged that I had never considered before-that was eye-opening." (Psychologist)

"Different perspectives helped me step outside my habitual way of thinking." (Physician)

Personal emotional impact and self-reflection: Many professionals experienced unexpected emotional engagement and personal insights.

"Without realizing it, I felt emotionally healed by the process." (Nurse)

"I started with anxiety about my role but ended up deeply immersed in listening." (Physician)

Structuring the Essential Experience (Professionals)

Among professionals, the essential structure of their experience in Open Dialogue was marked by a temporary shift in identity, as they stepped away from their traditional roles as experts and engaged in conversation as co-participants. This shift was experienced as both liberating and uncomfortable - freeing in its break from hierarchical norms, yet disorienting in its challenge to established professional boundaries. Letting go of control and embracing the open-ended nature of the dialogue stood in contrast to their usual problem-solving approaches. Engaging with multiple perspectives prompted reflexivity and encouraged professionals to rethink their assumptions. For many, the experience was personally meaningful, offering unexpected emotional insight and even a sense of healing. However, these moments were interwoven with ongoing tensions between spontaneity and structure. Facilitators, in particular, grappled with the challenge of maintaining neutrality while providing enough structure to sustain meaningful dialogue, revealing the complex skill set required for effective dialogical practice in professional contexts.

## Discussion

This study explored participants’ experiences in the Machiken Dialogue, an adaptation of Open Dialogue applied to everyday community worries. This study did not measure psychological or social outcomes such as resilience quantitatively; instead, it explored participants’ subjective experiences - how they felt safe, reflective, empathetic, and heard - within dialogical encounters. By analyzing qualitative responses from both general citizens and professionals, we identified key themes that highlight the potential benefits and challenges of structured dialogue in a non-clinical setting. Based on participants’ qualitative narratives, the Machiken Dialogue was perceived to create a sense of psychological safety, encourage self-reflection, enhance empathy through exposure to multiple voices, and highlight the value of silence and reflection in communication. These findings illustrate participants’ subjective experiences rather than measurable outcomes, consistent with the phenomenological aim of this study to explore lived meanings rather than quantify effects. These results align with previous research on Open Dialogue in psychiatric contexts, demonstrating its broader applicability in community-based interventions [1,2,7].

A primary finding of this study was the creation of a safe and accepting space where participants felt free to express their thoughts without fear of judgment. This is consistent with previous research emphasizing the importance of psychological safety in dialogical approaches [1,4]. The ability to share personal worries in an open yet structured format contributed to a sense of reassurance and social connectedness, which may help mitigate feelings of isolation [13]. Moreover, the iterative nature of reflective dialogues enabled participants to revisit and refine their thoughts, deepening their understanding of their own experiences and those of others.

Another notable theme was self-discovery through dialogue, where participants gained new insights into their emotions, biases, and personal growth. This aligns with the hermeneutic foundation of Open Dialogue, which posits that meaning is co-constructed through conversation [12]. Participants' reflections revealed that verbalizing their worries in a non-hierarchical space helped them observe their emotions more objectively, a process that has been documented in therapeutic Open Dialogue settings [7]. These findings suggest that the Machiken Dialogue provides a mechanism for introspection, which may contribute to emotional resilience and self-awareness beyond clinical applications.

The study also highlighted the impact of the multiplicity of voices and empathy. Exposure to different perspectives was frequently described as enriching, allowing participants to appreciate diverse viewpoints and deepen their capacity for empathy. This supports existing literature on polyphony in dialogue, which emphasizes that hearing and responding to multiple voices fosters mutual understanding [11]. By encouraging an open-ended conversational structure, the Machiken Dialogue facilitated deeper engagement with others' experiences, contributing to a stronger sense of community belonging. These findings reinforce previous research indicating that dialogical approaches can strengthen social networks and enhance collective resilience [13,23].

Additionally, the study revealed that silence and reflection played a crucial role in dialogue, allowing participants to process their emotions and thoughts more deeply. Initially perceived as uncomfortable, moments of silence became opportunities for attentive listening and thoughtful response. This insight highlights the reflective dimension of the Machiken Dialogue and underscores its contribution to fostering mindful, empathetic communication in both clinical and community contexts. This finding aligns with research suggesting that intentional pauses in conversation can encourage participants to articulate their thoughts more thoughtfully and attentively [8,24,25].

While the Machiken Dialogue showed promising benefits, several challenges were also identified, particularly concerning self-disclosure and facilitation. Some participants expressed initial hesitation in sharing personal worries, highlighting the delicate balance between openness and personal boundaries. This is a common challenge in dialogical approaches, as the level of disclosure varies among individuals based on their comfort and cultural norms [6]. Additionally, facilitators faced difficulties in maintaining neutrality while guiding discussions, underscoring the need for advanced training in dialogical facilitation. These findings suggest that ongoing facilitator training and support are crucial to ensuring the effectiveness of community-based dialogue interventions.

The responses from healthcare, welfare, and care professionals provided additional insights into the role of Open Dialogue in professional identity and practice. Many professionals reported experiencing a shift in their role, moving from an expert position to a more collaborative, participatory stance. While this transition was often described as liberating, some professionals expressed concerns about how their identity was perceived within the dialogue. Moreover, professionals noted difficulties in resisting the impulse to provide solutions, particularly when their training emphasized solution-oriented approaches. These findings resonate with previous studies on Open Dialogue implementation in psychiatric care, where professionals often struggle to balance structured guidance with the open-ended nature of dialogical practice [2,11]. Further research is needed to explore how professionals can best integrate dialogical principles into their practice while addressing role-related tensions.

While previous Open Dialogue studies have primarily focused on clinical and psychiatric contexts involving repeated meetings with patients and their families, the Machiken Dialogue represents a distinct adaptation for non-clinical, community-based use. Unlike the therapeutic Open Dialogue model, which emphasizes clinical recovery and symptom reduction, our dialogue sessions addressed everyday worries and community concerns among citizens and professionals who met only once. Despite this difference, the findings reveal that core dialogic mechanisms - such as polyphony, tolerance of uncertainty, and shared meaning-making - function similarly in fostering reflection, empathy, and connectedness.

This divergence highlights the adaptability of Open Dialogue principles beyond clinical environments. The Machiken Dialogue’s inclusive, single-session format enables broader participation and may serve as a bridge between professional and civic domains, supporting both emotional well-being and social cohesion. In this sense, it adds value by demonstrating how dialogical practice can be reframed as a community resource for mutual support and social connectedness, complementing traditional clinical applications of Open Dialogue.

A key strength of this study lies in its qualitative, phenomenological design, which enabled a nuanced understanding of participants’ lived experiences and the subtle processes that occur within dialogical encounters. By involving both citizens and professionals, the study captured a diversity of voices that illuminated how dialogic communication can bridge personal, professional, and civic boundaries. Methodologically, applying IPA to a non-clinical adaptation of Open Dialogue offers an innovative framework for exploring community communication processes.

In practical terms, the findings suggest that structured yet open dialogue can foster psychological safety, self-reflection, and empathy beyond psychiatric contexts. The Machiken Dialogue thus represents a model for cultivating reflective communication and mutual understanding in broader community and educational settings. These insights have implications for designing community-based health promotion, interprofessional education, and citizen-professional collaboration initiatives that emphasize empathy, shared meaning, and social connectedness.

Despite its contributions, this study has several limitations. First, the sample consisted of individuals who voluntarily participated in the Machiken Dialogue, which may introduce selection bias, as participants were likely predisposed to be open to dialogical engagement. Second, the qualitative nature of the study, while rich in depth, limits generalizability, and further quantitative studies could provide additional insights into the broader impacts of community-based Open Dialogue interventions. Third, while our analysis identified key themes, further longitudinal research is needed to assess the long-term effects of participation in structured dialogue sessions on emotional resilience and social connectedness.

Future research should explore the potential applications of Open Dialogue in other non-clinical settings, such as education, workplace environments, and conflict resolution. Additionally, comparative studies examining different facilitation methods may help refine best practices for implementing Open Dialogue in diverse cultural and social contexts. Expanding the scope of research beyond the Machiken Dialogue will provide a more comprehensive understanding of how dialogical approaches can be adapted to various community needs. Furthermore, integrating quantitative measures - such as validated scales of empathy, psychological safety, or reflective capacity - into future studies may complement the phenomenological insights and enhance the empirical grounding of dialogical research.

## Conclusions

This study highlights the potential of the Machiken Dialogue as an adaptation of Open Dialogue to address community concerns. Through phenomenological qualitative analysis, we identified key experiential themes, including psychological safety, self-discovery, empathy, and the significance of silence and reflection. Our findings suggest that structured dialogue interventions can foster personal insight, strengthen social support networks, and encourage reflective and empathetic communication among participants.

However, challenges related to self-disclosure, facilitation, and professional role transitions warrant further attention. As Open Dialogue continues to expand beyond psychiatric care, its application in broader community settings presents a promising avenue for enhancing social cohesion and emotional well-being. Further research is essential to refine and optimize the implementation of dialogical practices in diverse contexts, ensuring their effectiveness in fostering inclusive, supportive, and meaningful conversations.

## References

[1] Seikkula J, Alakare B, Aaltonen J, Holma J, Rasinkangas A, Lehtinen V (2003). Open dialogue approach: treatment principles and preliminary results of a two-year follow-up on first episode schizophrenia. Ethical Hum Sci Serv.

[2] Bergström T, Alakare B, Aaltonen J (2017). The long-term use of psychiatric services within the Open Dialogue treatment system after first-episode psychosis. Psychosis.

[3] Tribe RH, Freeman AM, Livingstone S, Stott JC, Pilling S (2019). Open dialogue in the UK: qualitative study. BJPsych Open.

[4] Olson M, Seikkula J, Ziedonis D (2014). The Key Elements of Dialogic Practice in Open Dialogue. https://www.umassmed.edu/globalassets/psychiatry/open-dialogue/keyelementsv1.109022014.pdf.

[5] Seikkula J, Arnkil TE, Eriksson E (2003). Postmodern society and social networks: open and anticipation dialogues in network meetings. Fam Process.

[6] Anderson H, Gehart DR (2022). Collaborative-Dialogic Practice: Relationships and Conversations That Make a Difference Across Contexts and Cultures. cultures.: Taylor & Francis.

[7] Freeman AM, Tribe RH, Stott JC, Pilling S (2019). Open Dialogue: a review of the evidence. Psychiatr Serv.

[8] Haarakangas K, Seikkula J, Alakare B, Aaltonen J (2007). Open dialogue: an approach to psychotherapeutic treatment of psychosis in northern Finland. Collaborative Therapy.

[9] Kadoma A, Kato M, Yamamoto M, Asano M, Shiraki K (2024). How do dialogical group sessions work for mothers of young children with parenting challenges in Japan?. Int J Qual Stud Health Well-being.

[10] Son D, Ishikawa H, Yonekura Y, Nakayama K (2023). The process of transformative learning in dialog café with health professionals and citizens/patients. J Prim Care Community Health.

[11] Arnkil TE (2018). Dialogical Meetings in Social Networks. https://www.taylorfrancis.com/books/mono/10.4324/9780429473685/dialogical-meetings-social-networks-tom-erik-arnkil.

[12] Bakhtin MM (2010). The Dialogic Imagination: Four Essays. https://utpress.utexas.edu/9780292715349/.

[13] Kinane C, Osborne J, Ishaq Y, Colman M, MacInnes D (2022). Peer supported Open Dialogue in the National Health Service: implementing and evaluating a new approach to mental health care. BMC Psychiatry.

[14] Mosse D, Pocobello R, Saunders R, Seikkula J, von Peter S (2022). Introduction: Open Dialogue around the world - implementation, outcomes, experiences and perspectives. Front Psychol.

[15] Rutledge E (2024). Open Dialogue: a rights-based approach to treatment in mental health care. Ir J Psychol Med.

[16] Seikkula J, Olson ME (2003). The open dialogue approach to acute psychosis: its poetics and micropolitics. Fam Process.

[17] Andersen T (1987). The reflecting team: dialogue and meta-dialogue in clinical work. Fam Process.

[18] Pietkiewicz I, Smith JA (2014). A practical guide to using interpretative phenomenological analysis in qualitative research psychology. Psychol J.

[19] Smith JA, Osborn M (2003). Interpretative phenomenological analysis. Qualitative Psychology: A Practical Guide to Research Methods.

[20] Heidegger M (1962). Being and Time. https://books.google.co.jp/books/about/Being_and_Time.html?id=S57m5gW0L-MC.

[21] Smith JA, Harré R, Van Langenhove L (1995). Idiography. Rethinking Psychology.

[22] Smith JA, Flowers P, Larkin M (2009). Interpretative Phenomenological Analysis: Theory, Method and Research. https://books.google.co.jp/books/about/Interpretative_Phenomenological_Analysis.html?id=UTxdBAAAQBAJ&redir_esc=y.

[23] Maude P, James R, Searby A (2024). The use of Open Dialogue in Trauma Informed Care services for mental health consumers and their family networks: a scoping review. J Psychiatr Ment Health Nurs.

[24] Hill CE, Thompson BJ, Ladany N (2003). Therapist use of silence in therapy: a survey. J Clin Psychol.

[25] Lane RC, Koetting MG, Bishop J (2002). Silence as communication in psychodynamic psychotherapy. Clin Psychol Rev.

